# Morphometric Reconstruction of Coronary Vasculature Incorporating Uniformity of Flow Dispersion

**DOI:** 10.3389/fphys.2018.01069

**Published:** 2018-08-29

**Authors:** Ravi Namani, Ghassan S. Kassab, Yoram Lanir

**Affiliations:** ^1^California Medical Innovations Institute Inc., San Diego, CA, United States; ^2^Faculty of Biomedical Engineering, Technion, Haifa, Israel

**Keywords:** coronary circulation, computer simulation, network reconstruction, morphometry, diameter assignment, perfusion dispersion

## Abstract

Experimental limitations in measurements of coronary flow in the beating heart have led to the development of *in silico* models of reconstructed coronary trees. Previous coronary reconstructions relied primarily on anatomical data, including statistical morphometry (e.g., diameters, length, connectivity, longitudinal position). Such reconstructions are non-unique, however, often leading to unrealistic predicted flow features. Thus, it is necessary to impose physiological flow constraints to ensure realistic tree reconstruction. Since a vessel flow depends on its diameter to fourth power, diameters are the logical candidates to guide vascular reconstructions to achieve realistic flows. Here, a diameter assignment method was developed where each vessel diameter was determined depending on its downstream tree size, aimed to reduce flow dispersion to within measured range. Since the coronary micro-vessels are responsible for a major portion of the flow resistance, the auto regulated coronary flow was analyzed in a morphometry-based reconstructed 400 vessel arterial microvascular sub-tree spanning vessel orders 1–6. Diameters in this subtree were re-assigned based on the flow criteria. The results revealed that diameter re-assignment, while adhering to measured morphometry, significantly reduced the flow dispersion to realistic levels while adhering to measured morphometry. The resulting network flow has longitudinal pressure distribution, flow fractal nature, and near-neighboring flow autocorrelation, which agree with measured coronary flow characteristics. Collectively, these results suggest that a realistic coronary tree reconstruction should impose not only morphometric data but also flow considerations. The work is of broad significance in providing a novel computational framework in the field of coronary microcirculation. It is essential for the study of coronary circulation by model simulation, based on a realistic network structure.

## Introduction

The coronary network supplies oxygen-carrying blood to each myocyte in the myocardium to meet its metabolic demand. This task is assigned to the coronary network consisting of millions of vessels of different diameters which are embedded within the myocardium. Flow in the network is determined by three major factors: (1) The network tree-like structure of asymmetrically bifurcating arteries and respective collecting venous one; (2) The vessels' compliance which responds to external loading by the periodically contracting myocardium to affect the coronary lumen area and the blood flow; (3) The three major flow control mechanisms which act in concert to actively regulate the coronary arterial diameters, aimed to meet the myocardium metabolic demand (Feigl, 1983). The flow control mechanisms are myogenic one which responds to the local trans-vascular pressure (Johnson, 1980; Kuo et al., 1988, 1990a; Davis, 2012); shear (flow) mechanism which responds to the local wall shear of the flowing blood (Kuo et al., 1990b, 1995; Jones et al., 1995b); and the metabolic mechanism which responds to imbalance in oxygen supply/demand in the myocardium (Feigl, 1983; Kanatsuka et al., 1989).

Despite considerable progress in recent decades in understanding coronary flow, further advances are impeded primarily due to considerable difficulties in experimental measurement of flow features in the heart which are due to the significant movement during the cardiac cycle, the inaccessibility of deep myocardial layer vessels, the dynamic myocardium-vessel interaction (MVI), and the difficulties in controlling the levels of each flow regulation mechanism under *in vivo* conditions. Mathematical modeling can be a powerful tool to supplement experiments. Previous modeling studies considered either the network structure (Karch et al., 1999; Beard and Bassingthwaighte, 2000) or the vessel interaction with the contracting myocardium (Downey and Kirk, 1975; Spaan et al., 1981; Krams et al., 1989; Rabbany et al., 1989; Kresh et al., 1990; Manor et al., 1994; Zinemanas et al., 1994; Vis et al., 1997; Smith, 2004; Westerhof et al., 2006; Algranati et al., 2010), or only the flow regulation (Liao and Kuo, 1997; Cornelissen et al., 2002; Carlson et al., 2008).

On the network structure, the very large number of coronary vessels precludes the possibility of deterministic reconstruction of the network. Statistical data-base is thus the method of choice. Such morphometric data was collected for the pig coronary tree (Kassab et al., 1993) and served as a basis for a number of flow studies in reconstructed coronary trees (Beard and Bassingthwaighte, 2000; Smith, 2004; Kaimovitz et al., 2005, 2010). Reconstruction based on statistical morphometric data is, however, non-unique. It may, in addition, lead to unrealistic flow features. Our preliminary flow analysis in morphometry-based reconstructed trees revealed that the predicted flow did not match measured perfusion dispersion in the terminal arterioles, being significantly higher compared to data measured by radioactive microspheres (King et al., 1985; Austin et al., 1994; Mori et al., 1995) and molecular microspheres (Stapleton et al., 1995; Matsumoto and Kajiya, 2001). In the present study, it was hypothesized that additional flow related constraints should be applied in order to produce realistic simulated coronary trees. The need to add flow constraints arises since the statistical morphometric data-base (Kassab et al., 1993) contains vessel order connectivity and statistics of order dependent diameter and length, but no data on diameter and length connectivity (correlation). Since flow is predominantly affected by the vessels' diameters, being dependent on their fourth power (Poiseuille equation), diameter re-assignment was used for flow optimization.

In the present study, a flow-based diameter re-assignment method was developed. In addition to the measured network morphometric data, the model considers the vessel interaction with surrounding myocardium and active diameter regulation. The procedure developed here was previously applied in the analysis of coronary flow regulation (Namani et al., 2018). It was used without details of rationale, the method procedure, no comparison with other studies or proof of validity. Here, we present analysis of the methodology and rationale, proof of validity compared with measured flow characteristics, and supplemented by detailed sensitivity analysis to the model parameters.

In view of the excessive computational cost of simulating dynamic regulated coronary flow in the entire arterial tree (few million vessels), and given the dominance of the microvasculature in flow resistance and control (Feigl, 1983; Jones et al., 1995a; Zamir et al., 2015), the present analysis focused on a microvascular sub-tree spanning vessel orders 1–6. This network was pruned from our previously reconstructed whole arterial LCX tree (Kaimovitz et al., 2005). To test the study hypothesis, the new diameter re-assignment was implemented, and the resulting flow was analyzed and compared with measured features of coronary flow.

## Methods

The tree reconstruction procedure consisted of three stages. First, an arterial microvascular subtree was pruned from the reconstructed LCX tree (Kaimovitz et al., 2005). Next, the subtree auto regulated flow was numerically analyzed. Finally, based on the predicted terminal order 1 arteriole flows, vessel diameters were iteratively re-assigned. The aim was to achieve measured physiological level of flow dispersion which is defined as the variance around the mean level of the flows at pre-capillary vessels. The flow dispersion is quantified by the flow coefficient of variation (CV = Mean/SD), where Mean is the mean flow across all pre-capillary arterioles, and SD is the respective standard deviation.

### Reconstruction of the coronary tree

The measured characteristics of the pig coronary tree are connectivity of vessel orders, vessel length and diameter, and myocardial depth. The LCX arterial tree was stochastically reconstructed (Kaimovitz et al., 2005) based on such morphometric data (Kassab et al., 1993). A 400-vessel arterial microvascular subtree was pruned from the reconstructed LCX tree. The stem vessel is of order 6 and the tree contains all vessels down to the pre-capillary (order 1) arterioles. Based on the morphometric data (Kassab et al., 1993), the subtree has both bifurcations and trifurcations. The conductivity matrix (Table 1), an essential input to the flow analysis, was derived from the network structure as elaborated in the Appendix.

**Table 1:** The network topology in matrix form indicating each vessel neighbors.

		**Indices of connected vessel numbers**									
		**1**	**2**	**3**	**4**	**5**	**6**	**7**	**8**	**9**	**10**
**Vessel number**	1	X	X	X							
	2	X	X	X	X	X					
	3	X	X	X			X	X			
	4		X		X	X			X	X	
	5		X		X	X					X
	6			X			X	X			
	7			X			X	X			
	8				X				X	X	
	9				X				X	X	
	10					X					X

*The table depicts the network neighbors of the first 10 vessels in the network. The conductance matrix of the entire network was obtained from this topology matrix*.

### Flow in a single coronary vessel

**Figure 1:**
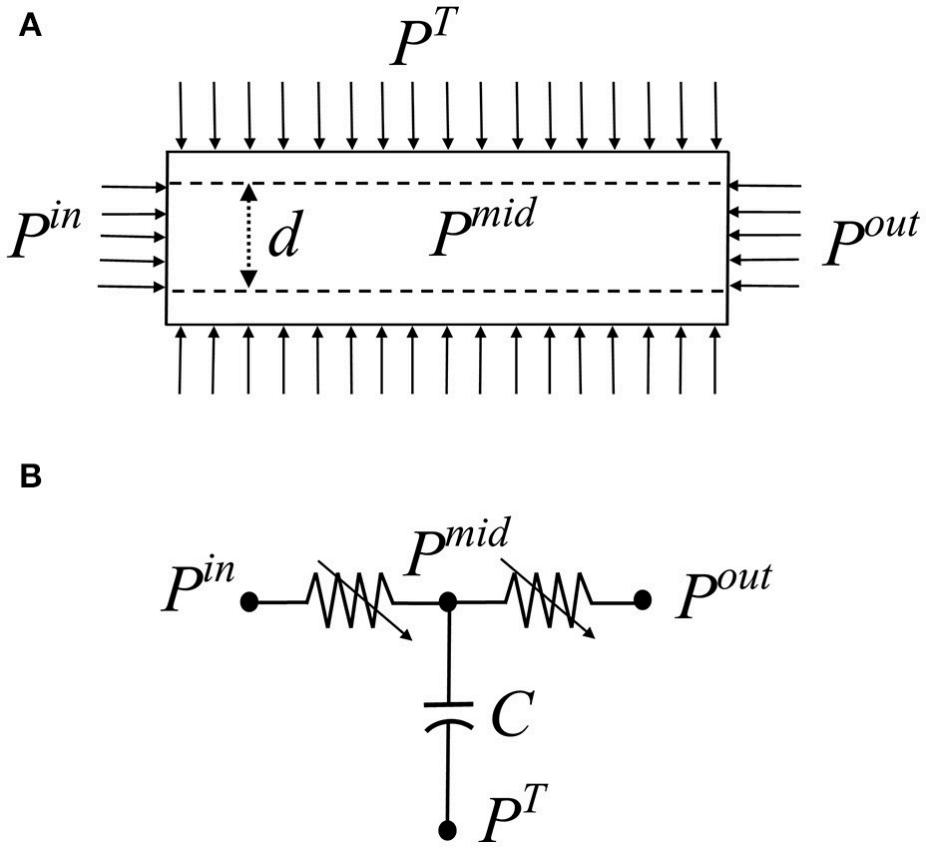
**(A)** Schematic of a single coronary vessel and the applied dynamic pressure boundary conditions. *P*^*in*^ is the inlet pressure, *P*^*out*^ is the outlet pressure, *P*^*T*^ is the tissue pressure exerted by the myocardium, *P*^*mid*^ is the unknown intravascular pressure. **(B)** The vessel 3-element Windkessel model comprising two resistors R_1_, R_2_ and a capacitive element C that accounts for the vessel volume changes.

In the coronary microvasculature, flow is determined predominantly by viscosity forces under given driving pressures, while the effect of inertia is negligible. Under this condition, conservation of momentum in a single cylindrical vessel (Figure 1A) yields the Poiseuille relation:

(1) $$\begin{array}{l} q\left(t\right)=\frac{\pi {\left[r\left(t\right)\right]}^{4}\Delta P_{L}\left(t\right)}{8\mu \left(r\right)L} \end{array}$$

where *q*(*t*) is the flow rate, *r*(*t*) is the vessel dynamic radius, Δ*P*_*L*_(*t*) is the longitudinal pressure drop, μ(*r*) is the dynamic viscosity which is a function of the vessel radius, and *L* is the vessel length. An equivalent validated non-linear 3-element Windkessel model (Jacobs et al., 2008) was used here for each vessel in lieu of Equation 1 (Figure 1B). The network model was assembled from such vessel models.

### Boundary conditions

Apart from the inlet and outlet pressures [*P*^*in*^*(t)* and *P*^*out*^*(t)*], the boundary conditions affecting the coronary flow are the myocardial vessel interaction (MVI) which is the myocardial loading on each vessel during the heartbeat, *P*^*T*^(*t*), consisting of the sum of three contributions. The first two are the intra-myocardial pressure (IMP) and the shortening-induced intra-myocyte pressure, *P*^*SIP*^*(t)* (Algranati et al., 2010). The third myocardial loading reflects the vessel radial traction by its tethering to the surrounding myocardium (Borg and Caulfield, 1979; Caulfield and Borg, 1979). IMP was taken to vary linearly with the myocardial depth (MRD) from zero at the epicardium to the dynamic LV cavity pressure *P*^*LV*^*(t)* in the endocardium.

The inlet pressure boundary condition *P*^*in*^*(t)*, adopted from measured pressures in order 6 arterioles (Chilian, 1991), was 87/55 mmHg in systole/diastole (average of 66 mmHg). The outlet pressure, *P*^*out*^*(t)* at a terminal order 1 arteriole was taken to depend on each vessel's myocardial relative depth (MRD) as predicted in a previous study under non-regulated conditions (Algranati et al., 2010). It was thus 82/−32 mmHg (average = 22 mmHg) in the endocardium and 34/0 mmHg (average = 13 mmHg) in the epicardium for systole/diastole. LV pressure signal, *P*^*LV*^(t), was taken to be 99/7 mmHg (average = 36 mmHg), as predicted from a distributive LV mechanical model (Nevo and Lanir, 1989) under a resting heart rate of 75 BPM. More details on the boundary conditions are presented in the Appendix.

### Diameter re-assignment

The basis for the diameter reassignment is the notion that given the vessel length, and assuming uniform flow in all terminal order 1 vessels, diameter of an upstream vessel should be compatible with a flow rate which is proportional to the number of terminal arterioles it feeds. Hence, from Poiseuille's formula (Equation 1), the vessel diameter *d* should follow as:

(2) $$\begin{array}{l} d=BX^{1/4},\;X=\frac{n_{pc}L}{\Delta P_{L}} \end{array}$$

where *n*_*pc*_ is the number of terminal order 1 arterioles in the crown of that vessel, *L* is the vessel length, and the pressure drop along the vessel length (Δ*P*_*L*_) is determined from the network flow analysis. The constant parameter *B* represents the target terminal arteriole flow *q*_*target*_ needed to balance the myocardium metabolic demand. Equations 1 and 2 yield for *B*

(3) $$\begin{array}{l} B={\left(\frac{128\mu \;q_{target}}{\pi }\right)}^{1/4} \end{array}$$

The target flow rate for the terminal arterioles *q*_*target*_ was set to be 0.4 × 10^−3^ mm^3^/s, within the measured range of resting diastolic flow which is 0.4–0.6 × 10^−3^ mm^3^/s for vessels of order 1 in dog hearts (Tillmanns et al., 1974; Ashikawa et al., 1986; Stepp et al., 1999). The dog heart level was adopted due to the absence of such data in the swine, and based on the remarkably similar relationship between left ventricle myocardial blood flow (LVMBF) and heart rate in dog and swine hearts (Figure 4 in Duncker and Bache, 2008). Diameter re-assignment was carried out under flow control by myogenic and shear regulations. Metabolic regulation was not included here based on the rationale that the low resting target flow rate represents a state in which the metabolic regulation is nearly inactive. The metabolic regulation plays a crucial role under higher metabolic demands. Diameter reassignment was an iterative procedure since the pressure drop along each vessel, Δ*P*_*L*_, changes each time diameters are modified. The re-assignment was subject to the constraints that the diameter level remains within the measured range of that vessel order (Kassab et al., 1993). If the diameter fell outside that range, the diameter was set to be at the limit of its order range. This was done to ensure the reconstructed network statistics to be compatible to that of the database (Kassab et al., 1993). In addition, re-assignment was restricted to comply with the constraint that a vessel diameter be smaller than its mother vessel (hydraulic continuity). If this condition was violated, diameter was assigned to be similar (difference was 0.1 μm) to that of the mother vessel. For order 0 vessels (capillaries), no correction was applied. Convergence of this iterative method was determined when the coefficient of variation in terminal arteriolar flows reduced to < 25%, i.e., within the range of measured data (Austin et al., 1994; Matsumoto and Kajiya, 2001).

#### Network flow solution and the diameters re-assignment

For a given state of the network vessels unloaded diameters, flow was iteratively solved under the dynamic pressure boundary conditions by imposing mass conservation in all the tree nodes at each time along the cardiac cycle. This yields a system of ordinary differential equations (ODEs). The method of solution is presented in detail in the Appendix. Briefly, for the given vessel unloaded radius, the loaded radius was taken to vary with the pressure difference across the vessel wall, which depends on the vessel's passive and active pressure-diameter relationships (PDR). The passive properties are the non-linear pressure-diameter relationships (Young et al., 2012) constrained by the vessel tethering to its surrounding myocardium. The active properties reflect the contractile and dilatory effects of the vessel's smooth muscle by myogenic (pressure-driven) and shear regulation mechanisms. Their contributions were taken from *in vitro* data on single isolated coronary micro-vessels (Liao and Kuo, 1997). Details are provided in the Appendix.

Once the network flow was solved for a given diameter state, each vessel unloaded diameter was iteratively re-assigned (by application of Equations 2, 3), and the network flow was solved following each diameter re-assignment until convergence was attained.

### Model predictions

In presenting the model results, emphasis will be on those predictions which can be compared with observed characteristics of the coronary network flow.

#### Longitudinal pressure distribution

Numerical solution of the network flow yields the intravascular pressures at each node and vessel in the tree. The associated pressure distribution can be contrasted with the few *in vivo* data that exist on the intravascular pressure in the coronary micro-vessels (Tillmanns et al., 1981; Chilian, 1991). In view of the paucity of coronary data, the predicted pressure distribution was also compared with parallel data in the cat mesentery (Fronek and Zweifach, 1975; Zweifach and Lipowsky, 1977), and in the hamster cheek pouch (Davis et al., 1986).

#### Spatial flow heterogeneity

As another test of validity of the diameter re-assignment methodology presented here, the model predicted flow dispersion in terminal arterioles was compared with data. This validation has three related but different aspects (Bassingthwaighte and Beyer, 1991; Yipintsoi et al., 2016). The flow dispersion level (CV), the flow fractal nature (self-similarity), and the flow spatial auto-correlation.

##### flow dispersion as a function of sample mass

Experimental evidence showed that flow dispersion decreases with increasing sample size (Bassingthwaighte et al., 1989; Matsumoto and Kajiya, 2001). In addition, the dispersion was found to have a fractal (self-similar) nature (Bassingthwaighte et al., 1989). When plotted on a log-log scale, self-similarity is seen as a linear dispersion vs. sample size relationship where the slope is 1–*D, D* being the fractal dimension (Bassingthwaighte et al., 1989). A value of *D* = 1.0 indicates highly correlated near neighbor flows and *D* = 1.5 indicates that neighboring flows are uncorrelated having spatial randomness. These observed characteristics of the coronary flow were examined in the predicted flow as another test of validity of the proposed diameter re-assignment. The tissue volume fed by a terminal arteriole was previously estimated to be 0.2–1.0 mm^3^ (Bassingthwaighte et al., 1974; Matsumoto and Kajiya, 2001). Since there are interspecies variations, the sample mass fed by a terminal arteriole was estimated in an independent manner. Based on the morphometric data of Kassab et al. (1993), approximately 10^6^ order 1 arterioles feed the 150 *gm* heart, which implies that each arteriole supplies approximately 0.15 *mg*. From this and the above listed estimates, the mass perfused by a terminal arteriole was set to be 0.2 *mg* in the present study. To estimate the dispersion vs. sample mass relationship, sample size was increased by grouping together tissue units supplied each by a single terminal arteriole. Sample size was increased up to 4.4 *mg*. The flow dispersion was represented by the coefficient of variation (CV = SD/Mean), SD being the standard deviation.

##### spatial autocorrelation of pre-capillary flows

Flow spatial autocorrelation is a measure of the flow continuity. The idea is to estimate the correlation of near-neighboring flows between regions as a function of the distance between them. The spatial location of vessels in 3-D space given by its x, y, z coordinates were used to calculate the Euclidean distance, *e* $=\sqrt{{\left(x_{1}-x_{2}\right)}^{2}+{\left(y_{1}-y_{2}\right)}^{2}+{\left(z_{1}-z_{2}\right)}^{2}}$, between any pair of terminal arterioles with spatial co-ordinates, (*x*_1_*, y*_1_*, z*_1_) and (*x*_2_*, y*_2_*, z*_2_). Per definition, the spatial autocorrelation function, *f(e)*, is the sum of co-variances in flow over all pairs of terminal arterioles separated by a distance *e*, divided by the flow variance over all terminal arterioles of the subtree. It is thus expressed as

(4) $$\begin{array}{l} f\left(e\right)=\frac{\underset{i,j}{\sum }\left(Q_{i}\left(e\right)-{\bar{Q}}_{term}\right)\left(Q_{j}\left(e\right)-{\bar{Q}}_{term}\right)}{n_{e}\;\cdot \;var\left(Q_{term}\right)} \end{array}$$

where *n*_*e*_ are the number of regions that are separated by a distance *e*. The regional flow was normalized by the total mean flow to derive relative flow *Q*. The regional relative flows are *Q*_*i*_(*e*) and *Q*_*j*_(*e*) in the *ith* and *jth* regions which are separated by a distance *e*, and *Q*_*term*_ is the flow in the pre-capillaries. Two aspects of the flow autocorrelation have been measured and modeled in previous studies. Accordingly, both the model predicted flow autocorrelation, and the predicted nearest-neighbor autocorrelation coefficient were estimated before and after diameter re-assignment and compared with data (Matsumoto and Kajiya, 2001) and with a previous model (Beard and Bassingthwaighte, 2000).

##### fractal structure of pre-capillary flows

The predicted autocorrelation function (Equation 4) was compared with a theoretical prediction of this function for terminal arteriole flows in a self-similar fractal tree structure (Beard and Bassingthwaighte, 2000). The spatial autocorrelation function of a fractal network with fractal dimension *D* as a function of the spatial distance *e* is given by

(5) $$\begin{array}{l} f\left(e\right)=0.5*\left[{\left(e+1\right)}^{4-2D}-2e^{4-2D}+{\left(e-1\right)}^{4-2D}\right] \end{array}$$

The spatial autocorrelation function *f(e)* that was determined from Equation 4 was plugged into Equation 5. Linear regression was carried out between *f(e)* and *e* and the exponent 4–2*D* was estimated, and hence *D*. This estimate of the fractal dimension *D* was compared with the *D* estimated from the dispersion-sample size analysis described above.

### Sensitivity analysis

The model parameters used for diameter re-assignment are those of the vessels' myogenic and flow regulations ρ_*m*_, ϕ_*m*_, *C*_*m*_, *F*_τmax_ (Appendix), the target terminal flow *q*_*target*_ and the network outlet pressure *P*^*out*^(Figure 1). Their values were adopted from published experimental studies (Liao and Kuo, 1997) and from simulation of non-active full sized network (Algranati et al., 2010). Since biological systems exhibit inherent variability, a sensitivity analysis was carried out by perturbing each parameter uniformly in all the network vessels. Each parameter was varied from the reference level. The perturbed levels of *q*_*target*_ were 0.2 and 0.6 × 10^−3^ mm^3^/s (the reference being 0.4 × 10^−3^ mm^3^/s). The outlet pressures *P*^*out*^ were perturbed by ±20 mmHg from their depth dependent reference levels. The activation parameters ρ_*m*_, ϕ_*m*_, *C*_*m*_, *F*_τmax_ were perturbed by ±25% from their reference levels. Upon each perturbation, diameter re-assignment and flow analysis were redone with the new set of parameters.

The sensitivity of the model predictions were assessed in relation to vessel diameters, terminal flow level and its dispersion (CV), flow fractal dimension estimated from both the dispersion and spatial autocorrelation of terminal flows, and network longitudinal pressure distribution.

## Results

### Flow effects of diameter re-assignment

**Figure 2:**
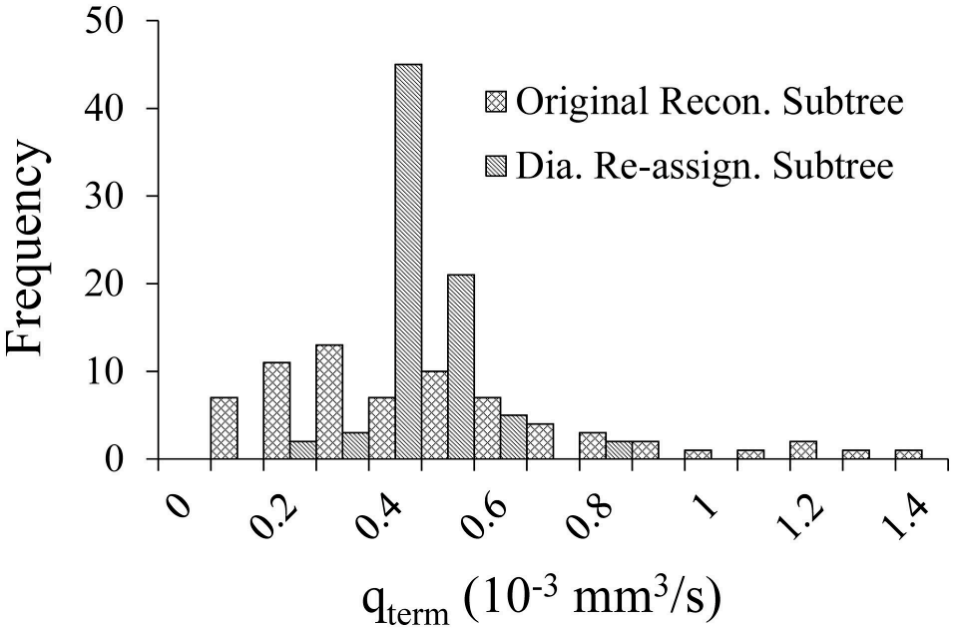
Comparison of the flow distribution in the terminal pre-capillary arterioles in the 400-vessel subtree under shear and myogenic controls prior vs. after diameter re-assignment. Each bin on the X-axis represents a 0.05 × 10^−3^ mm^3^/s range of terminal pre-capillary flows per cardiac cycle. The Y-axis is the pre-capillary frequency for each bin.

Diameter re-assignment significantly changed the frequency distribution of the terminal arteriole flow, *q*_*term*_ to be narrower (more uniform) with a higher peak close to the target flow of 0.4 × 10^−3^ mm^3^/s (Figure 2). The range of flows in the unmodified reconstructed tree was 0.04–2.57 × 10^−3^ mm^3^/s which changed after diameter re-assignment to be considerably narrower in the range of 0.14–0.73 × 10^−3^ mm^3^/s. In addition, in the native reconstructed network (prior to diameter re-assignment) the mean terminal flow was 0.57 × 10^−3^ mm^3^/s, while in the re-assigned network it reduced to 0.39 × 10^−3^ mm^3^/s which is within the physiological range. Terminal flow dispersion (CV) in the native reconstructed network was 0.97, and reduced in the diameter re-assigned network to be 0.22, which is within the physiological measured range (Table 2).

**Table 2:** Flow in the pre-capillary vessels under a target terminal flow of 0.4 × 10^−3^ mm^3^/s, before and after diameter re-assignment, in the passive state and under myogenic and shear flow regulations.

**Flow control**	**Method**	**Flow:** ***q_term_*** **(10^−3^ mm^3^/s)**	
		**(Mean + SD)**	**CV (SD/Mean)**
Myo + Shear.	Original Recon. Tree (Model)	0.57 ± 0.54	0.95
	Re-assigned Tree (Model)	0.39 ± 0.09	0.22
	Matsumoto et al., 1999 (Data)	–	0.18
	Austin et al., 1994 (Data)	–	0.20
Passive	Original Recon. Tree (Model)	1.63 ± 0.43	0.87
	Re-assigned Tree (Model)	1.26 ± 0.29	0.29
	Austin et al., 1994 (Data)	–	0.30

*The model predicted coefficients of variation (CV) is compared with published data*.

### Longitudinal pressure distribution

**Figure 3:**
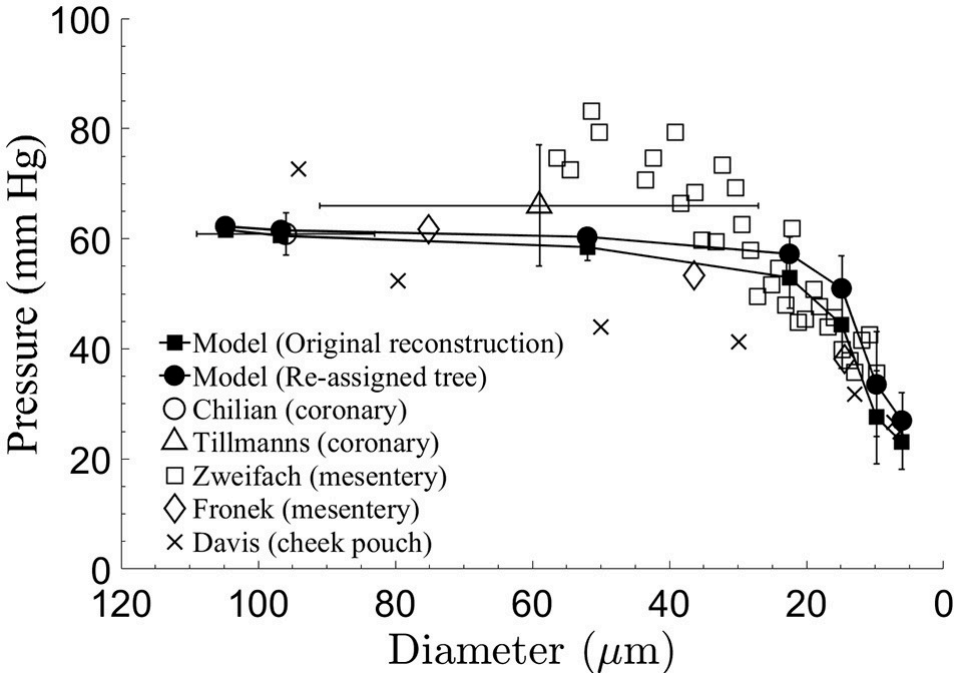
Comparison of the model-predicted time-averaged intravascular pressure distribution in the subtree after diameter re-assignment with the data sets of Chilian (1991) for pigs coronaries, of Tillmanns et al. (1981) for the epi-myocardium of rats and cats, of Fronek and Zweifach (1975) and Zweifach and Lipowsky (1977) for the cat mesentery, and of Davis et al. (1986), for the hamster cheek pouch. Model predictions and mesentery data show significant pressure drop in small micro-vessels.

The predicted pressure distribution (Figure 3) decreased from 62 mmHg in vessel order 6 to 27 mmHg in vessel order 1. The curve shows elevated pressure drop over the low vessel orders, with the highest drop in vessel orders 1–4. The pressures in these small vessels have the highest standard deviations. The current model predictions are compared with measured data. The comparison shows very good agreement with Chilian (1991) pig coronary data for order 6 vessels, and likewise with Tillmanns et al. (1981) rat and cat coronary data for a range of vessel sizes in the intermediate (orders 3,4,5) and order 6 vessels, as well as with Fronek and Zweifach (1975) cat mesentery data for intermediate vessel range. The cheek pouch data (Davis et al., 1986) differ substantially from the model predictions.

### Spatial flow heterogeneity

#### Flow dispersion as a function of sample mass

**Figure 4:**
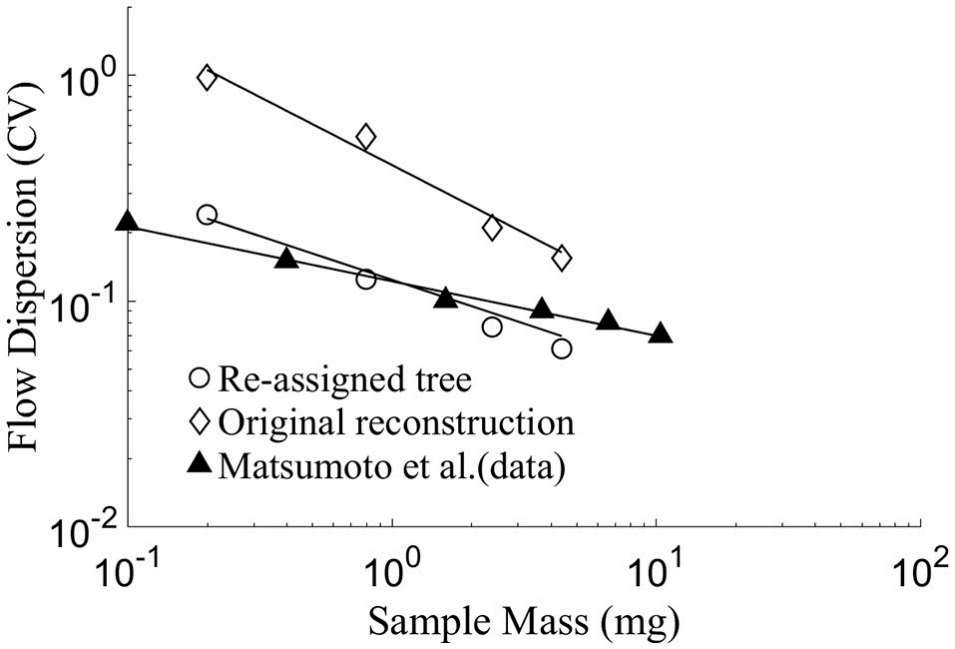
Log-log plot of model prediction and data of flow dispersion (represented by CV = SD/Mean) vs. perfused sample mass. The slope of the linear fit equals 1–D, where D being the fractal dimension (Bassingthwaighte et al., 1989). The results yield *D* = 1.44 for the diameter-reassigned tree, *D* = 1.62 for the original reconstructed tree, *D* = 1.24 for the data of Matsumoto et al. (1999) in the sub-endocardial region. There is a close agreement between the data of Matsumoto et al. and the present model prediction for the diameter re-assigned tree, while predictions of the original reconstructed tree yields a CV of up to an order of magnitude higher than the data.

The flow dispersion (CV) in myocardial regions was found to decrease exponentially (linearly in a log-log plot) with increase in sample tissue mass (Figure 4). In the native reconstructed tree prior to diameter re-assignment, the highest CV of 0.97 was found at the lowest sample mass of 0.2 *mg*, corresponding to the perfused mass by one terminal arteriole in the present study. The lowest CV of 0.16 was found at a sampled tissue mass of 4.4 *mg*. These values are one half to one order of magnitude higher than in the measured data of the rabbit ventricular free walls (Matsumoto et al., 1999). After diameter re-assignment, the CV reduced to 0.24 for a sample mass of 0.2 *mg*, which decreases linearly in the log-log plot to 0.06 for a sample mass of 4.4 *mg*. The re-assigned network flow dispersions vs. sample size relationship is in close agreement (Figure 4) with the data of Matsumoto et al. from rabbit's free ventricular wall (Matsumoto et al., 1999; Matsumoto and Kajiya, 2001). The regression of log(CV) vs. log (sample mass) is linear with a slope of −0.62 in the original reconstructed tree which changed to −0.44 after diameter re-assignment, in close agreement with the experimental data. These slope values yield fractal dimension *D* of 1.62 in the native reconstructed network and changed to 1.44 after diameter re-assignment.

#### Spatial autocorrelation of pre-capillary flows

**Figure 5:**
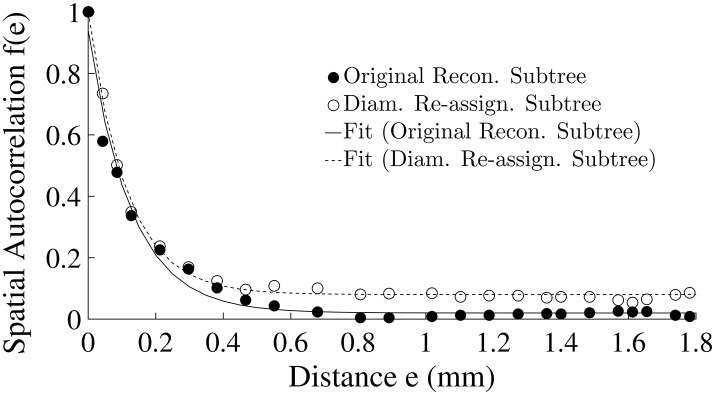
Flow spatial autocorrelation function between pairs of pre-capillary arterioles as a function of their spatial distance (Equation 4). Symbols are model estimates. The curves are single term exponentials of the form *a* · *exp* (−*e*/*e*_0_) + *c* fit to *f*(*e*) of the two subtrees. The parameter estimates are listed in Table 3.

Diameter re-assignment was found to significantly increase the spatial flow auto-correlation *f*(*e*) (Equation 4) at distances above ca. 0.4 mm (Figure 5) from an asymptotic average of 0.02 ± 0.01 for an unmodified tree to a level of 0.08 ± 0.01 after re-assignment. The function *f*(*e*) has an exponential decay with increasing spatial distance between terminal arterioles (Figure 5). A single term exponential of the form *a* · *exp*(−*e*/*e*_0_) + *c* was fit to *f*(*e*) for the subtrees prior and after re-assignment (Figure 5). The fit yielded parameter estimates (Table 3) showing that the most significant change induced by diameter re-assignment is a 300% increase of the long-range autocorrelation (from 0.02 to 0.08).

**Table 3:** Parameter of the spatial autocorrelation model $\hat{f}\left(e\right)=a\cdot exp\left(-e/e_{0}\right)+c\;$ estimated to fit the calculated spatial autocorrelation *f*(*e*) for the original reconstructed and the diameter re-assigned subtrees.

**Subtree**	***a***	***e_0_* (mm)**	***c***	***R^2^***
Original reconstructed	0.92	0.13	0.02	0.98
Diameter re-assignment	0.92	0.11	0.08	0.99

#### Fractal structure of pre-capillary flows

Estimates of the fractal dimension, *D* obtained from the flow dispersion analysis and from its auto-correlation are shown in Table 4. The difference in the estimates of *D* between the two methods is 4% for the unmodified reconstructed tree and 9% in the diameter reassigned tree. Both dispersion and spatial autocorrelation analyses gave a *D* > 1.5 in the original reconstructed sub-tree which means that flows are negatively correlated. In the diameter reassigned tree, *D* = 1.44 from the dispersion analysis and *D* = 1.31 from the spatial autocorrelation. Thus, the flow fractal nature of the network is maintained after diameter reassignment.

**Table 4:** Fractal dimension, D predicted from flow dispersion and from spatial autocorrelation analyses in a 400 vessel reconstructed coronary tree before and after diameter reassignment.

**Author**	**Type of study**	**Fractal dimension**, ***D***	
		**Original reconst**.	**Diameter reassign**.
Present work	Dispersion analysis	1.62	1.44
	Spatial autocorrelation	1.68	1.31
Karch et al., 2003	Model 1	1.45	
	Model 2	1.69	
Van Bavel and Spaan, 1992	Model	1.23	
Beard and Bassingthwaighte, 2000	Model	1.24	
Matsumoto et al., 1999; Matsumoto and Kajiya, 2001	Data	1.24	
Mori et al., 1995	Data	1.25	
Bassingthwaighte et al., 1989	Data	1.18–1.28	
Kleen et al., 1997	Data	1.39	
Iversen and Nicolaysen, 1995	Data	1.37	

*Comparison is listed with data and with other models which estimated D in artificially generated trees that were not based on morphometric measurements*.

### Sensitivity analysis

Two features are noticeable from the results of the diameter sensitivity analysis (**Table 6**). First, the diameter changes resulting from perturbations of the model parameters are small, <5%. The exceptions are the substantial diameter changes in order 6 vessels under changes in the following: (1) Outlet pressure *P*^*out*^, (2) increasing terminal target flow *q*_*target*_, (3) decreased myogenic parameter ρ_*m*_, and (4) under changes in the myogenic parameter *C*_*m*_. Likewise, the diameter changes are high in orders 5 and 6 vessels under reduction of the myogenic parameter ϕ_*m*_. The changes under decreased terminal target flow *q*_*target*_ are also > 5% even when consideration is given to the higher perturbation set for that flow (± 50% of the reference level) compared to the ±25% perturbations of the other parameters. Second, the trend of diameter change under a specific perturbation is different for the different vessel orders; i.e., diameter in some orders may increase while in others they decrease. The exceptions are the diameter responses to changes in the terminal target flow rate *q*_*target*_ which are positive in all orders under increased *q*_*target*_ and negative when it decreases, and corresponding changes under reduced *P*^*out*^ which are negative for all vessel orders.

Parameter perturbations affect the terminal flow rate *q*_*term*_ and its dispersion CV (**Table 7**). The terminal target flow rate *q*_*target*_ has the highest effect on the terminal flow, followed by that of the myogenic parameter ρ_*m*_. Any perturbation of *F*_τmax_ (i.e., both increase and decrease) changes the CV of the terminal flow in the same direction while for the other parameter opposite changes have opposite effects. The flow CV increases from its reference under all parameter perturbations except when *C*_*m*_ is reduced, where the highest effect on CV is that of the shear parameter *F*_τmax_, followed by those of the myogenic parameter ρ_*m*_ and the target flow rate *q*_*target*_.

Sensitivity analysis of the terminal flow fractal dimension *D* (Equation 5) to the model parameters (**Table 8**) shows that the two approaches of estimating the fractal dimension not only produce different values, but in addition predict at times the same trend of change in *D* while in other cases they predict a mutually opposite change. The dispersion analysis approach (left panel) predicts, in most cases, an increase in *D* values under perturbations, which in few cases crosses the boundary *D* = 1.50 between positive and negative correlation of near neighbor flows (Bassingthwaighte and Beyer, 1991). In contrast, the spatial autocorrelation estimated *D* (right panel in **Table 8**) is mostly lower than its dispersion estimated value except in two cases–reduced ρ_*m*_ and increased *F*_τmax_. In the latter case, the estimated (*D* = 1.65) is well within the negative correlated near neighbor flows.

**Figure 6:**
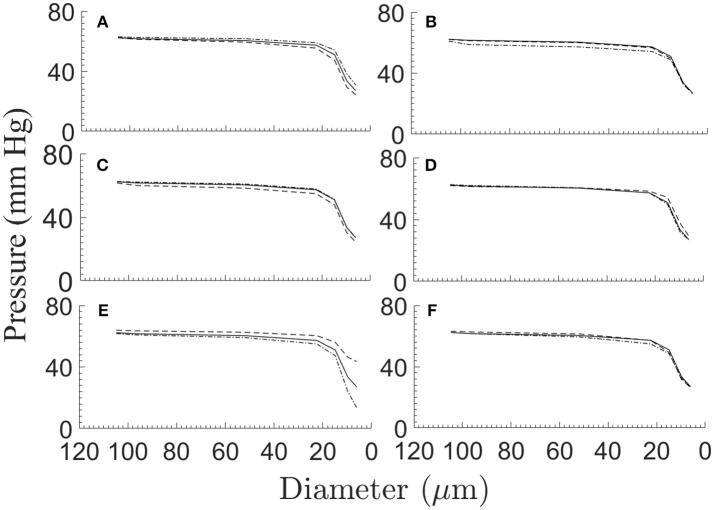
The network longitudinal (downstream) pressure distribution under ±25% perturbations of the myogenic and flow parameters ρ_*m*_ **(A)**, ϕ_*m*_ **(B)**, *C*_*m*_ **(C)**, and *F*_τmax_**(D)**, under ± 20 mmHg variations of the network outlet pressure *P*^*out*^**(E)**, and under ±50% variations of the terminal target flow rate *q*_*target*_ **(F)**. Depicted in each sub-figure are the pressure distribution in the reference case (solid line), and under positive (broken line) and negative (dash-dot line) perturbation.

Perturbations in the model parameters seem to have a small influence on the longitudinal (downstream) pressure distribution (Figure 6). For the effects of the myogenic and shear parameters ρ_*m*_, ϕ_*m*_, *C*_*m*_ and of *F*_τmax_ (Figures 6A–D), these insensitivities may stem from the uniform percent perturbations of the respective parameters across all vessel orders. The outlet pressure *P*^*out*^ has a small effect on the pressures in the smallest vessels (order 1 and 2) which are close to the network outlet, but has insignificant influence on the higher order vessels (Figure 6E). The target terminal flow rate *q*_*target*_ has a very small effect on the pressure distribution (Figure 6F).

## Discussion

This study addresses a longstanding difficulty in modeling coronary flow: how to reconstruct realistic coronary trees which mimic native trees in both structure and flow. Such reconstructions are indispensable for analyzing the coronary circulation. To this end, physiological constraints were proposed in conjunction with measured morphometric data. The present study used previous reconstructed coronary trees (Kaimovitz et al., 2005) which were built based on morphometric data (Kassab et al., 1993), and incorporated a new diameter re-assignment procedure designed to reduce flow dispersion to within measured ranges, without affecting the vessel orders or their lengths. The results show that application of this flow constraint provided reconstructed trees that conform with the morphometric data and at the same time, yield realistic flows which correspond with measured data in terms of the longitudinal pressure distribution, flow dispersion, spatial autocorrelation, and fractal structure (Austin et al., 1994; Matsumoto and Kajiya, 2001).

The current network reconstruction is the first which considers a comprehensive set of physiological aspects of the coronary circulation: measured morphometric-based tree-like structure of asymmetrically bifurcating vessels (Kassab et al., 1993); vessel compliance which allows for the external loading by the periodically contracting myocardium to affect the vessel lumen area and the blood flow; dynamic coronary flow driven by the likewise dynamic inlet and outlet pressures, myocardium-vessel interaction (Algranati et al., 2010); and the three major flow control mechanisms which act in concert to actively regulate the vessel diameters to meet the myocardium metabolic demand (Feigl, 1983).

A number of previous network flow studies considered morphometric data of vascular dimensions in symmetric vascular trees. Liao and Kuo (1997) applied a symmetric four element arterial network representing small arteries and large, intermediate, and small arterioles. Cornelissen et al. (2002) considered a network which consisted of 10 compartments in series where the first nine represented the arterial tree with resistance depending on the local pressure, and the distal one lumped the capillaries and the venules. Based on data of Van Bavel and Spaan (1992) and (Kassab et al., 1993, 1994; Kassab and Fung, 1994), Vis et al. (1997) applied a model network of symmetric bifurcating arterial and venous networks of 19 generations each, connected by a capillary network of parallel vessels. Arciero et al. (2008) and Carlson et al. (2008) network includes seven compartments connected in series, each consisting of identical parallel segments subjected to the same hemodynamic and metabolic conditions. They represent the upstream artery, the large arterioles, small arterioles, capillaries, small venules, large venules, and veins. The models of Schreiner's group (Schreiner and Buxbaum, 1993; Karch et al., 1999, 2003) generated 2D and 3D coronary networks by the method of constrained constructive optimization by successively adding new terminal segments while maintaining a set of physiological constraints. The actual network structure of asymmetrical bifurcating tree was considered by very few studies. Smith (2004) analyzed the flow in a discrete anatomically accurate model of the largest six generations of the coronary arterial tree. Beard and Bassingthwaighte (2000) network was reconstructed based on Kassab et al. (1993) morphometric data supplemented by self-similarity and avoidance algorithm. Kaimovitz et al. (2005, 2010) developed a large scale 3D reconstruction of the entire porcine coronary vasculature (arterial, capillaries, and veins) based on the morphometric data of Kassab et al. (1993, 1994) and subject to both global constraints relating to the location of the larger veins, and to local constraints of measured morphological features. No previous reconstruction considered flow constraints associated with the coronary flow dynamics, with the associated myocardium-vessel interaction (MVI), and included the active flow regulation.

As expected, flow dispersion in the myocardial regions was modified by diameter re-assignment (Table 2, Figure 4). A novel finding is that diameter re-assignment based on perfusion homogeneity consideration has dramatic effect on the dispersion and fractal nature of the flow, endowing it with dispersion/sample mass relationship and fractal nature that are similar to those of native *in vivo* coronary networks. These results suggest that the flow fractal nature is a consequence of not only the network asymmetry (Bassingthwaighte et al., 1989), but is also determined by the vessel diameters.

The results in Table 2 show that flow dispersion in the terminal pre-capillary arterioles is highly sensitive to diameter re-assignment. This sensitivity is attributed to the fact that by Poiseuille relationship (Equation 1), the rate of flow depends on the fourth power of diameter. Hence, diameter re-assignment is a powerful scheme for achieving target flow features. In addition, the developed scheme can be readily extended to incorporate other physiological features such as different profiles of non-uniform pre-capillary pressure distribution (Keelan et al., 2016) and a transmural non-uniform myocyte metabolic demand (Namani et al., 2018).

The pressure gradient along the subtree drives the flow. *In vivo* pressure data in the intermediate and small arterioles below order 6 that could serve for comparison with the model results are not available for the swine coronary micro-vessels. Data in these vessels are available for the cat mesenteric arterioles (Fronek and Zweifach, 1975; Zweifach and Lipowsky, 1977). Model comparison with these data shows close match with the intravascular pressures predicted by the model (Figure 3). The small differences between the slopes of the longitudinal pressure distribution curves between the model and mesenteric data may result from the difference in the vessel boundary conditions, in particular, the dynamic myocardial-vessel interaction (MVI) which is not present in the mesenteric microcirculation.

Flow dispersion in the terminal arterioles of the reconstructed and diameter re-assigned trees were found to increase with a decrease in sample size, similar to the trend observed experimentally by microsphere deposition (Bassingthwaighte et al., 1989). The log-log relation between flow dispersion and sample mass is linear (Figure 4) in both the model and measured data (Bassingthwaighte et al., 1989; Mori et al., 1995; Matsumoto and Kajiya, 2001). At sample sizes similar to the model ones, flow dispersion data are available (Matsumoto and Kajiya, 2001). There is close agreement between these data and the model predicted dispersions in the terminal arterioles. This stems from the realistic tree structure and from inclusion of the effects of myocardial vessel interaction and flow regulation in the present model, while not all of these factors were considered in previous models. This favorable comparison of the present model with data provides a rationale for using the diameter reassignment for reconstruction of coronary trees in future studies and offers flow dispersion as a new criterion for validation of these coronary trees.

The model predicted spatial auto-correlation of near neighboring flows, was found to decrease exponentially with the distance (Figure 5), a trend similar to that observed by microsphere experiments (Austin et al., 1994; Mori et al., 1995; Bassingthwaighte et al., 2012; Yipintsoi et al., 2016). Diameter re-assignment induced a significant increase of the long-range autocorrelation (Table 3) which suggests that distant flows are more correlated. This can be accounted for by recalling that re-assignment produces more uniform flows with a narrower dispersion range (Figure 2).

Our model estimates for nearest-neighbor autocorrelation coefficient of terminal arterioles flows are 0.47 and 0.52 (Table 5) for the original reconstructed and for the re-assigned sub-trees, respectively. These figures are comparable to the experimental value of 0.4 using a similar sample mass (0.2 mg) (Matsumoto and Kajiya, 2001), and close to some experimental estimates using higher sample masses (Mori et al., 1995; Yipintsoi et al., 2016).

**Table 5:** The nearest neighbor autocorrelation coefficients in the present model, and in several experimental data and other models.

	**Nearest neighbor autocorrelation**	
Present model	0.47	Original Reconst. Subtree (*m* = 0.2 mg)
	0.52	Dia. Re-assigned Subtree (*m* = 0.2 mg)
Experimental data	0.40	Matsumoto and Kajiya, 2001 (*m* = 0.2 mg)
Other models	0.37–0.43	Beard and Bassingthwaighte, 2000 (*m* = 16 mg–0.25 g)

Although, sample sizes used in previous studies were mostly different from the present model, the closeness of most reported coefficients supports the fractal nature of the network flow. In fractal networks the nearest-neighbor autocorrelation coefficient is independent of the sample size (Beard and Bassingthwaighte, 2000).

In models of the coronary tree, Beard and Bassingthwaighte (2000) found the nearest neighbor correlation coefficient to be in the range of 0.38–0.43 which is close to the range predicted by the present model.

The fractal dimension *D* is an established parameter for quantifying the global heterogeneity of coronary blood flow. It can be estimated either from the flow dispersion or from the spatial auto-correlation. Dispersion analysis estimated *D* to be 1.44 after diameter re-assignment. Spatial autocorrelation analysis gave an estimate for *D* of 1.31 which is closer to most of the measured data (Table 4). In contrast, prior to re-assignment, *D* was estimated to be 1.62 (> 1.5) which represents a tree with negatively correlated flows (*f*(*e*) < 0). This was rarely observed, and mainly in abnormal physiological conditions such as under reduced perfusion pressure (Mori et al., 1995) and stenotic conditions (Kleen et al., 1997). These results suggest that diameter re-assignment produces coronary flows that are fractal in nature and are comparable to *in vivo* observations; i.e., a fractal dimension of *D* < 1.5 with positive correlated near neighboring flows.

In comparison with previous models, the present model predicted *D* = 1.31 from spatial autocorrelation analysis of the diameter re-assigned tree is close to the model estimated values by Karch et al. (2003) (*D* = 1.38 – 1.45), by Van Bavel and Spaan (1992) (*D* = 1.23) and by Beard and Bassingthwaighte (2000) (*D* = 1.24) (Table 4).

Diameter sensitivity analysis of the model parameters (Table 6) reveals that the effects are mostly small. This counter-intuitive outcome is likely due to the large effect of the diameter on the vessel resistance to flow (Equation 1). The other finding (Table 6), is that the direction of diameter changes (increasing vs. decreasing) is mostly inconsistent, which seems to be also counter-intuitive. This is likely due to the diameter change by the re-assignment results from a complex non-linear interaction between the network asymmetric structure, the order-dependent passive and active mechanical properties, and the blood flow.

**Table 6:** Sensitivity of the network re-assigned vessel diameters in each order to perturbations in the model active parameters ρ_*m*_, ϕ_*m*_, *C*_*m*_, *F*_τ max_ (±25% of their reference), network output pressure *P*_*out*_ (±20 mmHg of its references), and the target flow rate q_target_ (±50% of its reference 0.4 × 10^−3^ mm^3^/s).

**Parameter and perturbation**		**Vessel diameter**, ***d*** **(**μ**m)**					
		**Order 1**	**Order 2**	**Order 3**	**Order 4**	**Order 5**	**Order 6**
Reference		9.6 ± 1.5	14.7 ± 1.3	21.9 ± 1.5	49.2 ± 4.4	99.9 ± 3.1	120.9 ± 0.0
*ρ_*m*_*	High	9.9 ± 1.2	14.3 ± 1.4	21.2 ± 1.7	46.7 ± 5.7	94.6 ± 11.0	115.8 ± 0.0
	*% Δd*	3.6	−2.9	−3.4	−5.1	−5.3	−4.3
	Low	9.3 ± 1.7	15.1 ± 1.0	22.5 ± 0.9	52.0 ± 0.0	101.7 ± 0.0	137.8 ± 0.0
	*% Δd*	−2.8	2.7	2.5	5.6	1.8	13.9
*φ_*m*_*	High	9.6 ± 1.5	14.6 ± 1.3	21.7 ± 1.7	49.0 ± 4.5	101.7 ± 0.0	114.4 ± 0.0
	*% Δd*	0.4	−0.7	−0.9	−0.4	1.8	−5.4
	Low	9.7 ± 1.3	14.1 ± 1.3	21.7 ± 1.4	48.3 ± 5.6	91.5 ± 9.2	103.9 ± 0.0
	*% Δd*	1.3	−0.9	−1.0	−1.9	−8.4	−14.1
*C_*m*_*	High	9.8 ± 1.3	14.5 ± 1.4	21.4 ± 1.6	47.9 ± 5.8	92.6 ± 7.9	102.4 ± 0.0
	*% Δd*	2.4	−1.8	−2.1	−2.7	−7.3	−15.3
	Low	9.6 ± 1.5	14.7 ± 1.3	21.8 ± 1.6	49.1 ± 4.4	101.7 ± 0.0	148.3 ± 0.0
	*% Δd*	0.2	−0.5	−0.6	−0.2	1.8	22.6
*F_τ*max*_*	High	9.3 ± 1.8	15.3 ± 0.7	22.7 ± 0.0	52.0 ± 0.0	101.7 ± 0.0	123.6 ± 0.0
	*% Δd*	−2.9	3.7	3.7	5.6	1.8	2.2
	Low	9.8 ± 1.2	14.3 ± 1.4	21.3 ± 1.6	47.9 ± 5.8	96.1 ± 8.4	123.8 ± 0.0
	*% Δd*	2.9	−2.6	−2.9	−2.8	−3.8	2.3
*P_*out*_*	High	10.2 ± 1.0	14.9 ± 1.1	22.1 ± 1.0	48.9 ± 5.0	97.4 ± 6.7	136.5 ± 0.0
	*% Δd*	6.4	1.0	0.8	−0.8	−2.5	12.9
	Low	9.2 ± 1.6	14.1 ± 1.5	20.6 ± 1.9	46.4 ± 5.8	94.1 ± 10.1	111.1 ± 0.0
	*% Δd*	−3.6	−4.1	−5.8	−5.8	−5.8	−8.1
*q_*target*_*	High	10.3 ± 0.9	15.4 ± 0.1	22.7 ± 0.0	52.0 ± 0.0	101.7 ± 0.0	202.6 ± 0.0
	*% Δd*	7.9	4.5	3.7	5.6	1.8	67.5
	Low	8.7 ± 1.6	12.6 ± 1.6	17.7 ± 2.0	38.8 ± 5.6	79.5 ± 10.4	101.8 ± 0.0
	*% Δd*	−9.3	−14.4	−19.3	−21.1	−20.5	−15.8

%Δ*d = (d−d_reference_)/d_reference_*.

The results presented in Table 6 allow comparison of the effects of the myogenic and flow regulations on the vessel diameters. The results show that the effects of their perturbations (ρ_*m*_, *F*_max_, respectively) on the network vessel diameters are small and of approximately comparable levels. In addition, it is seen that the effects of these perturbations on order 1 vessel diameter is generally opposite to their effects on other vessel orders. This result may be accounted for if one recalls that at *in vivo* conditions, the highest effect of the metabolic regulation is on order 1 vessels since the metabolic conducted response is highest at the interface of capillaries with these vessels and drops exponentially toward higher order vessels (Namani et al., 2018).

An interesting conclusion from the results in Table 7 is that under virtually all perturbations, the flow dispersion (CV) is higher than the reference level (= 0.22). The basis and implications of this prediction requires further inquiry.

**Table 7:** Sensitivity of the terminal flow rate and its dispersion over the terminal order 1 vessels to perturbations in the model active parameters ρ_*m*_, ϕ_*m*_, *C*_*m*_, *F*_τmax_ (±25% of their reference), network output pressure *P*^*out*^ (±20 mmHg of its reference), and the target flow rate q_target_ (±50% of its reference 0.4 × 10^−3^ mm^3^/s).

**Parameter**	**Perturbation**	***q_*term*_* × 10^−3^ mm^3^/s**	**CV**
Reference		0.39 ± 0.09	0.22
*ρ_*m*_*	High	0.31 ± 0.10	0.32
	Low	0.50 ± 0.16	0.33
*φ_*m*_*	High	0.45 ± 0.10	0.23
	Low	0.33 ± 0.09	0.26
*C_*m*_*	High	0.42 ± 0.12	0.28
	Low	0.41 ± 0.09	0.22
*F_τ*max*_*	High	0.30 ± 0.13	0.42
	Low	0.35 ± 0.12	0.34
*P^*out*^*	High	0.30 ± 0.11	0.37
	Low	0.43 ± 0.10	0.24
*q_*target*_*	High	0.67 ± 0.25	0.37
	Low	0.21 ± 0.06	0.30

It is not surprising that with the increase in the terminal flow dispersion (CV), the flow fractal dimension *D* changes as well (Table 8). Most affected is the *D* estimates from the dispersion vs. sample mass relationship, which in several cases changes the flow from being spatially positively correlated (*D* < 1.50) to being negatively correlated (*D* > 1.50).

**Table 8:** Sensitivity of the terminal flow fractal dimension D to perturbations in the model active parameters ρ_*m*_, ϕ_*m*_, *C*_*m*_, *F*_τmax_ (±25% of their reference), network output pressure *P*_*out*_ (±20 mmHg of its reference), and the target flow rate q_target_ (±50% of its reference 0.4 × 10^−3^ mm^3^/s).

**Parameter**	**Perturbation**	**Fractal dimension** ***D*** **derived from**	
		**Dispersion analysis**	**Spatial autocorrelation**.
Reference		1.44	1.31
*ρ_*m*_*	High	1.53	1.29
	Low	1.40	1.50
*φ_*m*_*	High	1.56	1.32
	Low	1.45	1.35
*C_*m*_*	High	1.49	1.33
	Low	1.48	1.21
*F_*max*_*	High	1.25	1.65
	Low	1.46	1.36
*P_*out*_*	High	1.50	1.39
	Low	1.55	1.45
*q_*target*_*	High	1.51	1.18
	Low	1.56	1.43

## Limitations of the study

A limitation of the study is that the vessel re-assigned diameters and resulting flow in them depend on the level of the target terminal flow (Equation 3). The applied target flow level was selected from measured data of resting metabolic demand. Another limitation is the size of the network analyzed, which although consisting of 400 vessels is relatively small compared with the full-sized network. Applying the current diameter re-assignment methodology to the entire coronary tree is needed since it will allow for more accurate pressure boundary conditions based on measured data. Otherwise, the network size should have no effect on the diameter re-assignment, nor on the flow in each vessel, provided the appropriate pressure boundary conditions of inlet, outlet and tissue pressure (MVI) are applied. The rationale is that the flow in any subtree chosen is determined by the vessels dimensions and by the inlet, outlet and tissue pressure, and is otherwise independent of flow events in other branches. Finally, consideration of additional physiological criteria to the flow dispersion, such as myocardial metabolic heterogeneity, may provide a more accurate diameter re-assignment. Such considerations can be readily incorporated to the methodology developed here.

## Conclusion

A diameter re-assignment method was developed and applied on morphometry-based reconstructed coronary networks, with the goal to obtain realistic coronary flow characteristics. This method focuses on the size of each vessel crown and the number of terminal pre-capillary vessels it supplies. The results show that whereas flow features in the original reconstructed subtree (without diameter re-assignment) significantly differ from measured data, application of diameter re-assignment was found to improve the physiological realism of the flow field in terms of correspondence with measured data of the longitudinal pressure distribution and of the terminal flow dispersion, spatial autocorrelation, and fractal structure. These results suggest that future coronary flow studies using reconstructed vascular networks should apply both morphometric data and flow considerations to reconstruct the network. This conclusion likely applies (with appropriate modifications) to circulation studies in skeletal muscle, and perhaps in other tissues as well.

## Author contributions

RN, YL, and GK made significant contributions to creation and design of the study, interpretation of data, and writing the manuscript. RN and YL developed the algorithms. RN wrote the code, ran the simulations and collected the data. RN, YL, and GK approve the final copy of the manuscript, agree to be responsible for all aspects of the research in the manuscript and confirm the role of authorship.

### Conflict of interest statement

The authors declare that the research was conducted in the absence of any commercial or financial relationships that could be construed as a potential conflict of interest.
