# Supplementary material for: Morphometric Reconstruction of Coronary Vasculature Incorporating Uniformity of Flow Dispersion
Source: Front Physiol. 2018 Aug 29;9:1069. doi: 10.3389/fphys.2018.01069 (PMC6123366; doi:10.3389/fphys.2018.01069)
Supplement: Supplementary file 1 [file Data_Sheet_1.pdf]

Appendix

The inputs to the model are the tree structure, vascular properties and pressure boundary conditions. The flow rates in the pre-capillaries are determined from network flow analysis described below. As the flow regulation is a highly nonlinear phenomenon that occurs over several cardiac cycles, it is linearized by assuming the active vessel properties to vary only between cardiac cycles and do not vary within a cardiac cycle. The regulated variables of diameters, pressures and shear stresses in the tree at the end of each cardiac cycle are from the network flow analysis which then update the microvascular properties.

A.1 Lumped model of the coronary tree

Lumped models for flow in each vessel was based on an electric-hydraulic analogy and were formulated with a resistor-capacitor (RC) element. The R-C circuit has two nonlinear resistors ($\Re_1$, $\Re_2$) connected in series with one capacitor *C*. Flow in each vessel was solved by a three element Windkessel lumped model (Jacobs, 2008). The capacitive element represents the volume change in each elastic vessel. The resistance $\Re$ (or conductance *G*) and capacitance *C* of each vessel are:

$$\Re(t) = 2\Re_1(t) = 2\Re_2(t) = \frac{8\mu(r)L}{\pi r(t)^4} \;, \qquad G(t) = 1/\Re(t) \tag{A1}$$

$$C(t) = \frac{dV}{d\Delta P} = 2\pi r(t) L \frac{dr(t)}{d\Delta P} \tag{A2}$$

The junction common to the resistors and the capacitor has an unknown pressure, P^{mid}. The junction of a vessel bifurcation has an unknown nodal pressure (P^{node}). Based on the conservation of fluid mass, the net flow at both the junctions is zero. Assumptions in the Windkessel model are *a)* vessel geometry is uniform, *b)* flow in the vessels is laminar, *c)* blood viscosity in all the vessels is a nonlinear function of vessel diameter (Pries et al., 1994) , *d)* active and passive vessel properties are spatially homogeneous in each vessel, *e)* the dynamic extravascular pressure P^T is uniform along each vessel but varies between vessels depending on their trans-mural location, and *f)* the active vessel response depends on the time averaged pressure, flow and metabolic signal.

A system of Ordinary Differential Equations (ODEs) were derived based on the conservation of mass for all junctions as,

$$\Delta q_i + \frac{dV_i}{dt} = \frac{\Delta P_i(t)}{\Re_i} - \frac{d}{dt}\left[\pi\left(R_i\right)^2 L_i\left(P_i^{mid} - P_i^T\right)\right] = 0 \;; \quad i = 1,2,3... \tag{A3}$$

where P_i^T is the given input signal of the extra-vascular pressure which depends on the myocardial transmural wall location. The mass conservation in each vessel R-C junction is:

$$\frac{\Delta P_{L,i}(t)}{\Re_i} = C_i \frac{d\left(P_i^{mid}(t) - P_i^T(t)\right)}{dt} \tag{A4}$$

where $\Delta P_{L,i}(t)$ is longitudinal pressure drop in each vessel.

The net flow between mother and daughter vessels at a designated "network node" is zero. Application of mass conservation at each node, additional equations for the nodal pressures were obtained. For an ith vessel, which is neither source nor sink, mass balance at the vessel inlet yields:

$$\left(P_{i,n_{-1}}^{mid}-P_i^{in}\right)G_{i,n_{-1}}+\left(P_i^{mid}-P_i^{in}\right)G_i+\left(P_{i,n_{-2}}^{mid}-P_i^{in}\right)G_{i,n_{-2}}=0 \tag{A5}$$

Hence, the inlet nodal pressure as a function of neighboring vessel pressures and conductance is given by:

$$P_i^{in}\left(t\right)=\frac{P_i^{mid}G_i+P_{i,n_{-1}}^{mid}G_{i,n_{-1}}+P_{i,n_{-2}}^{mid}G_{i,n_{-2}}}{G_i+G_{i,n_{-1}}+G_{i,n_{-2}}} \tag{A6}$$

Similarly, applying mass balance at the outlet node for the ith vessel gives:

$$P_i^{out}\left(t\right)=\frac{P_i^{mid}G_i+P_{i,n_1}^{mid}G_{i,n_1}+P_{i,n_2}^{mid}G_{i,n_2}}{G_i+G_{i,n_1}+G_{i,n_2}} \tag{A7}$$

For a source vessel, the inlet pressure, $P^{in}(t)$ and for a sink vessel, the outlet pressure, $P^{out}(t)$ are prescribed boundary conditions. The governing equations are assembled for the network into a system of ODEs. The coupled ODEs are nonlinear, time dependent and are represented in matrix form as:

$$\frac{d\boldsymbol{P^{mid}}}{dt}=\boldsymbol{AP^{mid}}+\boldsymbol{B} \tag{A8}$$

For a coronary tree with ***n*** number of vessels, ***A*** is a square matrix of size ***n*** x ***n*** and ***B*** is an ***n*** x 1 vector.

A.2 Construction of A and B matrices

The network structure matrix in Table 1 for a subtree (n = 400) was used to build the coefficient matrices ***A*** (n x n) and ***B*** (n x 1). The connectivity matrix is an n x n square matrix with the row index of a filled cell indicating the vessel number and the column index indicating the vessel in direct contact. The indices of the non-zero elements at an ith row in ***A*** correspond to the ith vessel properties and its neighbors. A vessel connected to bifurcating vessels at its origin and end has indices (i, n_{-1}), (i, n_{-2}), (i, n_1) and (i, n_2) for the mother, sister and two daughters respectively. The corresponding conductance's are: $G_{i,n_{-1}}, G_{i,n_{-2}}, G_{i,n_1}, G_{i,n_2}$. A vessel with a trifurcating branch at its end has an additional daughter with index (i, n_3) with conductance $G_i^{n_3}$. A vessel with a trifurcating vessel at its origin has an additional sister with index (i, n_{-21}). For a vessel, i, connected to bifurcations at both its ends, the elements of matrices ***A*** are

$$\frac{dP_i^{mid}}{dt} = A_{i,n_{-1}} P_{i,n_{-1}}^{mid} + A_{i,n_{-2}} P_{i,n_{-2}}^{mid} + A_{i,i} P^{mid} + A_{i,n_1} P_{i,n_1}^{mid} + A_{i,n_2} P_{i,n_2}^{mid} + B_i \tag{A9}$$

$$A_{i,i} = \frac{2G_i}{C_i}\left(\frac{G_i}{G_i + G_{i,n_{-1}} + G_{i,n_{-2}}} + \frac{G_i}{G_i + G_{i,n_1} + G_{i,n_2}} - 2\right),\ A_{i,n_{-1}} = \frac{2G_i}{C_i}\left(\frac{G_{i,n_{-1}}}{G_i + G_{i,n_{-1}} + G_{i,n_{-2}}}\right) \tag{A10}$$

$$A_{i,n_{-2}} = \frac{2G_i}{C_i}\left(\frac{G_{i,n_{-2}}}{G_i + G_{i,n_{-1}} + G_{i,n_{-2}}}\right),\ A_{i,n_1} = \frac{2G_i}{C_i}\left(\frac{G_{i,n_1}}{G_i + G_{i,n_1} + G_{i,n_2}}\right),\ A_{i,n_2} = \frac{2G_i}{C_i}\left(\frac{G_{i,n_2}}{G_i + G_{i,n_1} + G_{i,n_2}}\right) \tag{A11}$$

For a vessel, *i*, connected to a bifurcation at its origin and a trifurcation at its end, the elements of matrices ***A*** are

$$\frac{dP_i^{mid}}{dt} = A_{i,n_{-1}} P_{i,n_{-1}}^{mid} + A_{i,n_{-2}} P_{i,n_{-2}}^{mid} + A_{i,i} P^{mid} + A_{i,n_1} P_{i,n_1}^{mid} + A_{i,n_2} P_{i,n_2}^{mid} + A_{i,n_3} P_{i,n_3}^{mid} + B_i \tag{A12}$$

$$A_{i,i} = \frac{2G_i}{C_i}\left(\frac{G_i}{G_i + G_{i,n_{-1}} + G_{i,n_{-2}}} + \frac{G_i}{G_i + G_{i,n_1} + G_{i,n_2} + G_{i,n_3}} - 2\right) \tag{A13}$$

$$A_{i,n_1} = \frac{2G_i}{C_i}\left(\frac{G_{i,n_1}}{G_i + G_{i,n_1} + G_{i,n_2} + G_{i,n_3}}\right),\ A_{i,n_2} = \frac{2G_i}{C_i}\left(\frac{G_{i,n_2}}{G_i + G_{i,n_1} + G_{i,n_2} + G_{i,n_3}}\right) \tag{A14}$$

For a vessel, *i*, connected to a trifurcation at its origin and a bifurcation at its end, the elements of matrices ***A*** are

$$A_{i,i} = \frac{2G_i}{C_i}\left(\frac{G_i}{G_i + G_{i,n_{-1}} + G_{i,n_{-2}} + G_{i,n_{-21}}} + \frac{G_i}{G_i + G_{i,n_1} + G_{i,n_2}} - 2\right) \tag{A15}$$

$$A_{i,n_{-1}} = \frac{2G_i}{C_i}\left(\frac{G_{i,n_{-1}}}{G_i + G_{i,n_{-1}} + G_{i,n_{-2}} + G_{i,n_{-21}}}\right),\ A_{i,n_{-2}} = \frac{2G_i}{C_i}\left(\frac{G_{i,n_{-2}}}{G_i + G_{i,n_{-1}} + G_{i,n_{-2}} + G_{i,n_{-21}}}\right) \tag{A16}$$

If an ith vessel is terminating, the elements at each row in ***A*** are

$$A_{i,i} = \frac{2G_i}{C_i}\left(\frac{G_i}{G_i + G_{i,n_{-1}} + G_{i,n_{-2}}} - 2\right),\quad A_{i,n_{-1}} = \frac{2G_i}{C_i}\left(\frac{G_{i,n_{-1}}}{G_i + G_{i,n_{-1}} + G_{i,n_{-2}}}\right) \tag{A17}$$

$$A_{i,n_{-2}} = \frac{2G_i}{C_i}\left(\frac{G_{i,n_{-2}}}{G_i + G_{i,n_{-1}} + G_{i,n_{-2}}}\right), \tag{A18}$$

For the source vessel, the elements of ***A*** are

$$A_{1,1} = \frac{2G_1}{C_1}\left(\frac{G_i}{G_1 + G_{1,n_1} + G_{1,n_2}} - 2\right), \qquad A_{1,n_1} = \frac{2G_1}{C_1}\left(\frac{G_{i,n_1}}{G_1 + G_{1,n_1} + G_{1,n_2}}\right), \text{ and}$$

$$A_{1,n_{-2}} = \frac{2G_1}{C_1}\left(\frac{G_{1,n_2}}{G_1 + G_{1,n_1} + G_{1,n_2}}\right) \tag{A19}$$

For all interior vessels, the elements of **B** are

$$B_i = \frac{dP_i^T}{dt} \tag{A20}$$

For a pre-capillary i^{th} vessel, the elements at each row in ***B*** are

$$B_i = \frac{dP_i^T}{dt} + \frac{2G_i}{C_i}P^{out} \tag{A21}$$

For the source vessel, the elements of ***B*** are

$$B_1 = \frac{dP_1^T}{dt} + \frac{2G_1}{C_1}P^{in} \tag{A22}$$

For a single vessel, boundary conditions at the inlet and outlet are

$$P_1^{out}(t) = P_2^{in}(t) = P_3^{in}(t) = P^{node}(t); P_1^{in} = P^{in}; P_2^{out} = P_3^{out} = P^{out} \tag{A23}$$

A.3 The flow boundary conditions

Waveforms of the inlet pressure, $P^{in}(t)$, outlet pressure, $P^{out}(t)$, LV pressure, $P^{LV}(t)$, and intra-myocyte pressure, $P^{SIP}(t)$, are input signals to the flow analysis (Fig. A.1). The $P^{out}(t)$ signal was interpolated for different transmural locations based on predictions from simulation of the unregulated flow in an entire coronary network which included arterial and venous trees and four identical representative capillary networks, at relative myocardial depths (MRD) of 0.125, 0.375, 0.625 and 0.875 (Algranati et al., 2010). P^{LV}(t) waveform was taken from predictions based on a preliminary study of a distributive LV mechanical model (data not shown) under resting heart rate of 75 BPM. Several considerations guided the choice of the $P^{in}(t)$ signal for the sub-endocardial 400 vessel network. The first is the pressure drop from the aorta to the trunk vessel (order 6) of the subtree. On the other hand, there is a pressure increase due to the added intra-myocyte pressure, $P^{SIP}(t)$ which develops during contraction (Rabbany et al., 1989). In addition, $P^{in}(t)$ must provide for sufficient flow perfusion in the terminal order 1 vessels (0.4 x 10^{-3} mm^3/s) (Tillmanns et al., 1974;Ashikawa et al., 1986;Stepp et al., 1999). Based on these considerations, $P^{in}(t)$ was chosen to be 87/55 mmHg (with average $\bar{P}^{in}$=66) in systole/diastole and the signal shape was adopted from Algranati et al. (Algranati et al., 2010). The network output pressure P^{out} signal was assigned for each terminal vessel to be between the previously predicted sub-epicardium and sub-endocardium signals P^{out}_{subepi} and $P^{out}_{subendo}$ (Fig. A.1), depending on the transmural location of the

vessel. The tissue pressure $P^T(t)$ signal was derived based on a previous analysis of un-regulated coronary flow (Algranati et al., 2010).

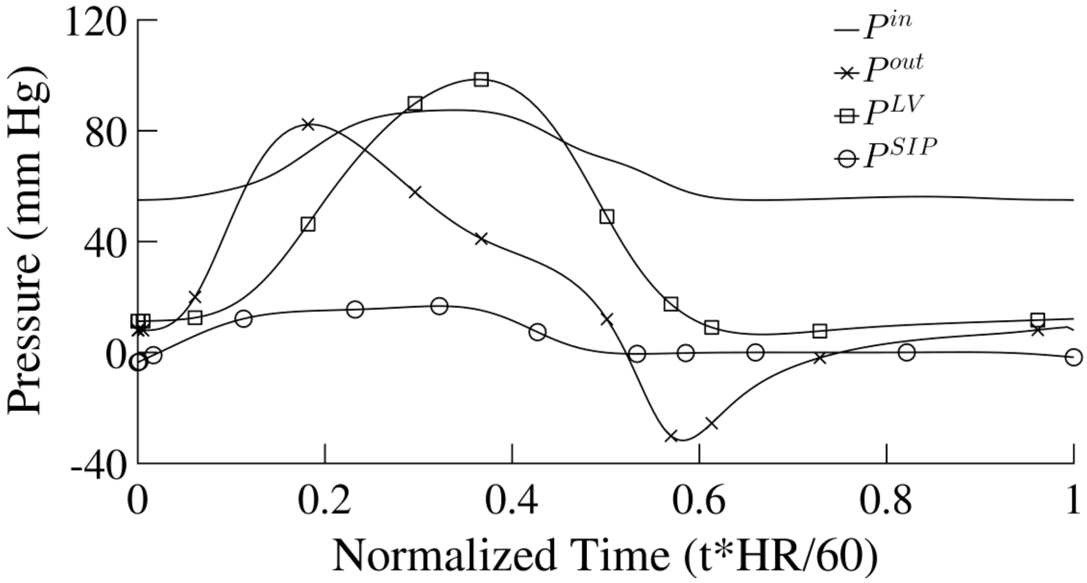

Fig A.1: *Signals of the periodic pressure boundary conditions.* $P^{in}(t)$*, the inlet pressure in the subtree trunk order 6 vessel is based on the measured range in these vessels (Chilian, 1991). The outlet pressure,* $P^{out}(t)$ *at a terminal order 1 arteriole was taken to be dependent on each vessel relative myocardial depth (MRD) (Algranati et al. 2010). The LV pressure* $P^{LV}(t)$ *was adopted from a distributive LV mechanical model (Nevo and Lanir, 1989) under a resting heart rate of 75 BPM.*

A.4 Vessel wall constitutive properties

The vascular wall is a composite of passive and active elements. The passive vascular model includes the vessel tethering to the myocardium. The active properties are due to the myogenic response of the smooth muscle cells and the flow regulation is due to the endothelial response to the blood near wall shear stress. The vascular mechanical properties are dependent on the vessel size.

The passive vessel tension is described by Laplace law as:

$$T_{pas} = \Delta P \cdot r_{reg} \tag{A24}$$

Vessels do not collapse due to the combined action of vessel tethering, passive response of vessel wall and active smooth muscle cell contraction. Hence, the balance of forces on the wall are given by

$$\Delta P \cdot r_{reg} + T_{teth} = T_{pas} + T_{act} \tag{A25}$$

The pressure generated by the tethering struts in the gap between vessel wall and myocardium is taken to be a quadratic function of the gap as in

$$P_{str}^{myo} = C_{str}(r_0 - r_{reg})^2 / r_0 \qquad \text{(A26)}$$

The strut pressure leads to the tethering tension, T_{teth} given as

$$T_{teth} = C_{str}(r_0 - r_{reg})^2 \qquad \text{(A27)}$$

The radius of a vessel under flow regulation is given as

$$r_{reg} = r_p - A\Delta r_m \qquad \text{(A28)}$$

where r_p , *A*, and Δr_m are respectively the vessel's passive radius, its flow-induced activation and the radius reduction due to the myogenic response.

Following Liao and Kuo (1997) experimental study and modeling, the shear vessel activation *A* is a sigmoidal function of the absolute value of time-averaged shear stress $|\bar{\tau}|$, and is given by

$$A = (1 - F_\tau), \quad F_\tau = F_{\tau max} \frac{|\bar{\tau}|}{K_\tau + |\bar{\tau}|} \qquad \text{(A29)}$$

Following the same experimental study, the passive radius, r_p and the change in vessel radius due to myogenic response, Δr_m are given respectively by

$$r_p = B_p + \frac{A_p - B_p}{\pi}\left(\frac{\pi}{2} + arctan\left(\frac{\Delta P - \phi_p}{C_p}\right)\right) \qquad \text{(A30)}$$

$$\Delta r_m = \frac{\rho_m}{\pi}\left(\frac{\pi}{2} - arctan\left(\left[\frac{\Delta\bar{P} - \phi_m}{C_m}\right]^{2m}\right)\right) \qquad \text{(A31)}$$

Order-dependent values of the above passive and active vessel parameters have been experimentally determined for some micro-vessel orders (Liao and Kuo, 1997) and interpolated for other orders (Namani et al., 2018).

The passive vessel tension is determined from the regulated radius as

$$\bar{T}_{pas} = \Delta P \cdot r_{reg} \qquad \text{(A32)}$$

The passive, tethering and active vessel stiffness's under flow regulation conditions are

$$k_{pas} = \frac{d\Delta P}{2\pi dr}$$
$$k_{teth} = \frac{C_{str}}{\pi}(R_0 - R_{reg}) \quad \text{(A33)}$$
$$k_{act} = C_1 T_{act} + C_0$$

The composite vessel dynamic stiffness is the sum of the three stiffness components given as

$$k = k_{act} + k_{pas} + k_{teth} \quad \text{(A34)}$$

From the total dynamic stiffness, the vessel compliance as a function of the dynamic trans-vascular pressure, $\Delta P(t)$ is

$$\frac{dr}{d\Delta P} = \frac{r_{reg}}{2\pi k - \Delta\bar{P}} \quad \text{(A35)}$$

The vessel compliance is a nonlinear function of the vessel dynamic radius. The vessel compliance is written as a Taylor's series expansion of the dynamic vessel radius and neglecting any second order variation in vessel radius leads to the following expression

$$r(t) \simeq r_{reg} + \frac{dr}{d\Delta P}\left(\Delta P(t) - \Delta\bar{P}\right) \quad \text{(A36)}$$

A.5 Numerical solution of network flow

The ODE is integrated over the cardiac cycle for each vessel as a function of time. The elements of ***A*** are vessel properties – conductance and capacitance given by Eqs. A10 – A19 and elements of ***B*** are Eqs. A20 – A22. Construction of ***A*** and ***B*** matrices and multiplication of the square matrix ***A*** with ***P*** is a significant computational challenge as Eq. A8 has to be evaluated for every time instant during the numerical integration process. The time span of integration for the system of ODE's was the time taken for one entire cardiac cycle. The cardiac time cycle was normalized to 1.0 and the system of ODE's was iteratively solved till the periodicity condition between successive cardiac cycles was satisfied. Periodicity condition is met when the difference in P^{mid} between the start and end of the cardiac cycle for each vessel did not vary by more than 0.005 Pa. Numerical accuracy of the solution is met when the net flow at the mid-point in all the vessels and at the network bifurcating or trifurcating nodes was less than 0.1%.

A.6 Validation of the sub-tree flow solution

To verify the accuracy of the numerical flow solution, simulations were carried out to verify if the flow periodicity condition was satisfied; i.e., smoothness of the transition between nodal pressures from the end of one cardiac cycle to the start of the next. The smoothness tolerance was set to 0.075 mmHg. The convergence of the computational results was estimated as follows: based on the network flow solution, the net inflow/outflow deviation at each time point (from the requisite zero level) was calculated at each vessel mid-node and at each network bifurcation. This was carried out for both the passive and regulated network flow, and under both steady as well as

dynamic flow conditions. Convergence was satisfied when all flow deviations were smaller than 1% of the inflow to the respective node and vessel junctions.

REFERENCES

Algranati, D., Kassab, G.S., and Lanir, Y. (2010). Mechanisms of myocardium-coronary vessel interaction. *Am J Physiol Heart Circ Physiol* 298**,** H861-873.

Ashikawa, K., Kanatsuka, H., Suzuki, T., and Takishima, T. (1986). Phasic blood flow velocity pattern in epimyocardial microvessels in the beating canine left ventricle. *Circ Res* 59**,** 704-711.

Liao, J.C., and Kuo, L. (1997). Interaction between adenosine and flow-induced dilation in coronary microvascular network. *Am J Physiol* 272**,** H1571-1581.

Namani, R., Kassab, G.S., and Lanir, Y. (2018). Integrative model of coronary flow in anatomically based vasculature under myogenic, shear, and metabolic regulation. *J Gen Physiol* 150**,** 145-168.

Pries, A.R., Secomb, T.W., Gessner, T., Sperandio, M.B., Gross, J.F., and Gaehtgens, P. (1994). Resistance to blood flow in microvessels in vivo. *Circ Res* 75**,** 904-915.

Rabbany, S.Y., Kresh, J.Y., and Noordergraaf, A. (1989). Intramyocardial pressure: interaction of myocardial fluid pressure and fiber stress. *Am J Physiol* 257**,** H357-364.

Stepp, D.W., Nishikawa, Y., and Chilian, W.M. (1999). Regulation of shear stress in the canine coronary microcirculation. *Circulation* 100**,** 1555-1561.

Tillmanns, H., Ikeda, S., Hansen, H., Sarma, J.S., Fauvel, J.M., and Bing, R.J. (1974). Microcirculation in the ventricle of the dog and turtle. *Circ Res* 34, 561-569.
